# Force Sensor Attachable to Thin Fiberscopes/Endoscopes Utilizing High Elasticity Fabric

**DOI:** 10.3390/s140305207

**Published:** 2014-03-12

**Authors:** Tetsuyou Watanabe, Takanobu Iwai, Yoshinori Fujihira, Lina Wakako, Hiroyuki Kagawa, Takeshi Yoneyama

**Affiliations:** 1 School of Mechanical Engineering, College of Science and Engineering, Kanazawa University, Ishikawa, Japan; E-Mails: linawakako@se.kanazawa-u.ac.jp (L.W.); kagawa@se.kanazawa-u.ac.jp (H.K.); yoneyama@se.kanazawa-u.ac.jp (T.Y.); 2 Department of Mechanical Engineering, College of Science and Engineering, Kanazawa University, Ishikawa, Japan; E-Mails: fuji3134@stu.kanazawa-u.ac.jp (T.I.); fuji3134@stu.kanazawa-u.ac.jp (Y.F.)

**Keywords:** force sensing, force visualization, panty stocking, endoscope

## Abstract

An endoscope/fiberscope is a minimally invasive tool used for directly observing tissues in areas deep inside the human body where access is limited. However, this tool only yields visual information. If force feedback information were also available, endoscope/fiberscope operators would be able to detect indurated areas that are visually hard to recognize. Furthermore, obtaining such feedback information from tissues in areas where collecting visual information is a challenge would be highly useful. The major obstacle is that such force information is difficult to acquire. This paper presents a novel force sensing system that can be attached to a very thin fiberscope/endoscope. To ensure a small size, high resolution, easy sterilization, and low cost, the proposed force visualization–based system uses a highly elastic material—panty stocking fabric. The paper also presents the methodology for deriving the force value from the captured image. The system has a resolution of less than 0.01 N and sensitivity of greater than 600 pixels/N within the force range of 0–0.2 N.

## 1. Introduction

The endoscope is becoming an increasingly important tool in the field of medicine. In physical examinations, endoscopes are a powerful tool for detecting diseases such as cancer. In medical operations, endoscopes allow practitioners to perform surgeries through small incisions, which can provide minimally invasive surgery and hence facilitate quick healing in patients. However, endoscopes do not give tactile/force information. Such information might be helpful for increasing the precision of examinations and improving the quality of treatments. In particular, tactile/force information is important when the visible area is limited. A typical example occurs in neurosurgery where the doctor is required to treat tumors that are seated deeply within the brain and surrounded by healthy tissues. Hence, the goal of this paper is to develop force sensors that can be attached to the tip of an endoscope so that the operator can investigate tissues seated deeply within organs.

Many force sensors have been developed for forceps and medical manipulators. Puangmali *et al.* [1] reviewed previously conducted researches of force and tactile sensors for minimally invasive surgery. The purpose of force sensing is to give feedback to the operator on the sensation of touching an organ, namely, force/haptic feedback (see [2–5] for reviews). Most force sensors that have been developed include electric devices such as strain gauges. However, electrical devices are not always suitable for medical devices. Electrical force sensors need amplifiers and wiring for signal transfer. This causes the size and cost of the overall system to become large. In addition, sterilizing or disinfecting such electrical devices is difficult. In special environments such as those of magnetic resonance imaging (MRI), the use of electricity should be avoided. One solution to these issues is to not use electrical parts such as wiring and circuits. Takaki *et al.* [6] developed a force sensor based on force visualization by using moiré fringe patterns. Tadano and Kawashima [7] developed a system to generate feedback on force sensation without a force sensor by utilizing a pneumatic servo system. Kawahara *et al.* [8] developed a system to measure the stiffness of an organ by pushing the organ using air and capturing the deformation using a camera. Peirs *et al.* [9] developed a force sensor that detects the deformation of a flexible structure by using optical fibers. Using fibers leads to issues pertaining to wiring, such as signal distortion resulting from bending, twisting, and chirping. Tada *et al.* [10] developed a force sensor that functions in MRI environments. A point light source is attached to tip of an elastic frame. If a force is applied to the elastic frame, the position of peak illumination changes. By detecting the change via photosensors, the applied forces can be estimated. These sensors are mainly used for laparoscopic surgery, and the size of the parts and the range of measurable forces are different. The part sizes and forces measured for laparoscopic surgery are larger (in the cm and N ranges, respectively) than the corresponding values that would be ideal for endoscopy in neurosurgery (in the mm and mN ranges) Sensors utilizing visualization of force have been developed, although the purpose of such sensors is not medical. Ohka *et al.* [11] have developed a three-axis force sensor by observing the states of conical feelers using a camera. Kamiyama *et al.* [12] have developed a sensor that can measure the direction, magnitude, and distribution of force by observing two layers of spherical markers using a camera. However, to apply these concepts to force sensors in small and thin fiberscopes, the sensor size should be reduced and a method to construct small markers must be developed. In general, this issue is considered to be the disadvantage of sensors utilizing force visualization, as mentioned in previous studies [5,13]. On the other hand, we previously developed a robotic system with force sensor and feedback systems for neurosurgery [14–16]. Unfortunately, the developed force sensor was based on a strain gauge system, and as a result, the above issues of sterilization and MRI compatibility were not resolved.

**Figure 1.**
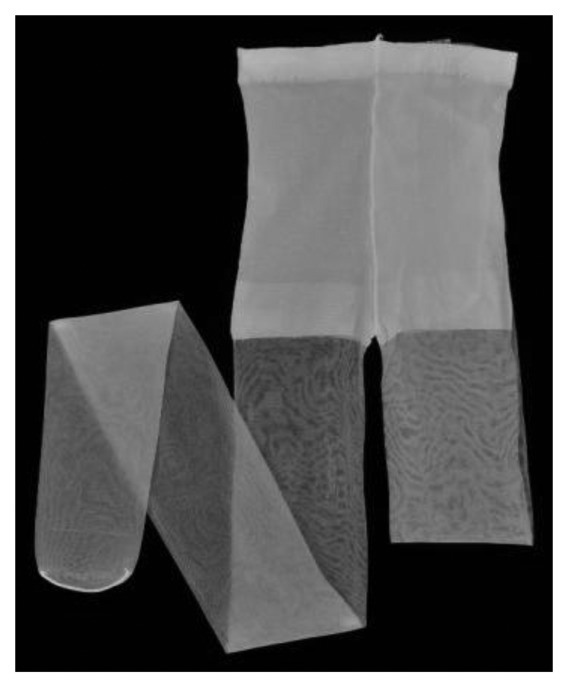
Panty stocking fabric.

With these issues in mind, this paper presents a novel force sensing system that is attachable to a very thin and commercially available fiberscope (diameter: 1.9 mm) with the goal of realizing force sensing in the deep areas of organs. The main contributions of the developed system are as follows:

*Force visualization mechanism utilizing a highly elastic panty stocking fabric*: We have developed a part that transforms force into deformation of panty stocking fabric (shown in Figure 1). This fabric is highly elastic and can deform even if the applied force is very small, as will be shown in Sections 3 and 4. Furthermore, this material is thin, inexpensive, and lightweight. The developed part can be attached to a fiberscope, and the deformation of the fabric can be captured using a camera.

*High resolution and small size*: Owing to the high elasticity of the stocking fabric, a high resolution of less than 0.01 N and a sensitivity of greater than 600 pixels/N are realized. The part used for detecting force is very compact, and the diameter of the entire system is less than 4 mm. It does not include electrical parts; therefore, sterilization is simple and MRI-compatibility easy to achieve. In addition, the part can be manufactured at a low cost and is disposable.

In a typical situation, a medical doctor examines the candidate area of a tumor by touching or nudging it with the developed sensor to find the affected area precisely, remove only the affected area, and minimize damage to the surrounding tissues, while observing the area with other endoscopes. In this case, the examination is normally performed by pushing tissues; examinations involving the release of tissues are rare. Therefore, this paper focuses on the case of pushing tissues. The measured force value is displayed on the monitor, superimposed on the image obtained by the endoscope. In this case, the feedback information is shown at the video rate. Therefore, high-sampling-rate data are not always needed, and video rate data are sufficient. Hence, image processing requiring relatively high computational effort can be adopted if the computing time is less than the video rate (10–30 Hz). This paper will thus use an endoscope for sensing force. Other benefits of using the endoscope are as follows. First, if a medical doctor has an endoscope, only the force visualization part is required for constructing a force sensor. Functional extension is then easy and can be done at a low cost. Second, as the endoscope is a conventional medical instrument, approval of the new system will be easy because only the force visualization part will need to be approved. Similarly, the sterilization procedure for the new system should be simple because we only need to consider how to sterilize the force visualization part. Finally, the proposed force sensor should be readily acceptable to medical doctors because it is an extended version of the commonly used endoscope.

The remainder of this paper is organized as follows: first, the structure of the part providing force visualization is presented. Next, the characteristics of the developed part (the relationship between the applied force and the corresponding deformation of the fabric) are evaluated. Subsequently, the method to derive the force value from the captured image is described and the validity of the presented system is shown via experimentation. Finally, the obtained results and outlook are summarized.

## 2. Structure of the Part Providing Force Visualization by Utilizing Stocking Fabric

### 2.1. Basic Principle for Force Sensing

The key to satisfying the requirements of easy sterilization and small size in force sensing is to remove the electronics in the sensor. One solution is force visualization. This involves transforming force into deformation and capturing the deformation as an image. The second requirement is a small range and high resolution. Using neurosurgery as an example, the typical magnitude of force in this application is around 0.1 N [14–16]. A resolution of less than 0.01 N is then required [14–16]. The solution for getting such a small range and high resolution is to use highly elastic panty stocking fabric.

In summary, we have developed a force visualization device that utilizes panty stocking fabric, has no electric parts, and is attachable to a fiberscope/endoscope. At the force visualization area, force is transformed into deformation of the high elastic fabric. By deriving the amount of deformation through the captured image, an applied force of small magnitude can be derived with high resolution.

### 2.2. Basic Structure of Part

**Figure 2.**
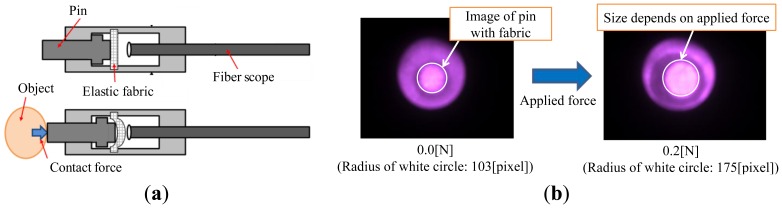
(**a**) Schematic structure of force visualization part. (**b**) Captured image of pin (with elastic fabric).

Figure 2a shows the basic structure for the force visualization part. Its main parts are a pin, fabric, the fiberscope, and their connecting parts. The pin slides along the depth of the fiberscope (horizontal direction in Figure 2a). The elastic fabric sits between the pin and the fiberscope. If objects or tissues make contact with the pin, the pin approaches the fiberscope. Because the pin is in contact with the elastic fabric, the distance it moves depends on the magnitude of applied/contact force. Namely, the elastic fabric plays the role of a spring. In the fiberscope, the pin covered with the elastic fabric is observed. Figure 2b shows an example of the captured image. The circle around the center is the image of the pin with the fabric. If the applied/contact force is large, the radius of the circular image is also large. The magnitude of the applied/contact force can then be derived from the radius of the circular image of the pin.

**Figure 3.**
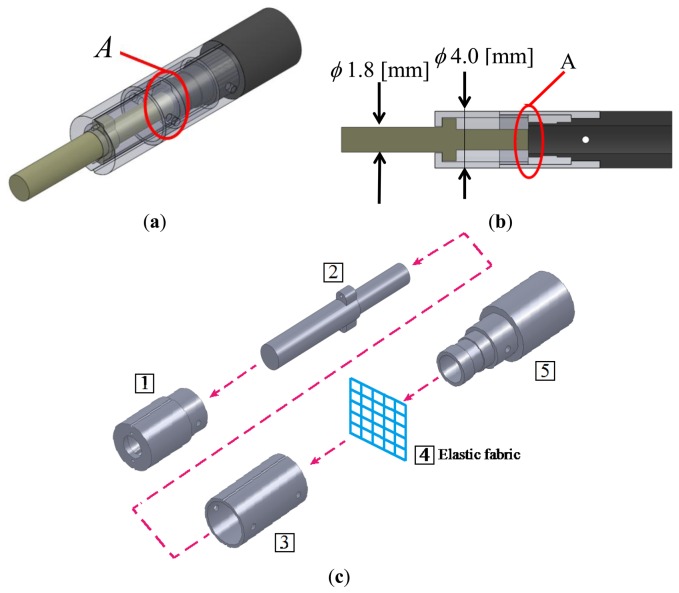
(**a**) Design of force visualization part: three-dimensional view (**b**) Design of force visualization part: sectional view (**c**) Overview of assembly process.

**Figure 4.**
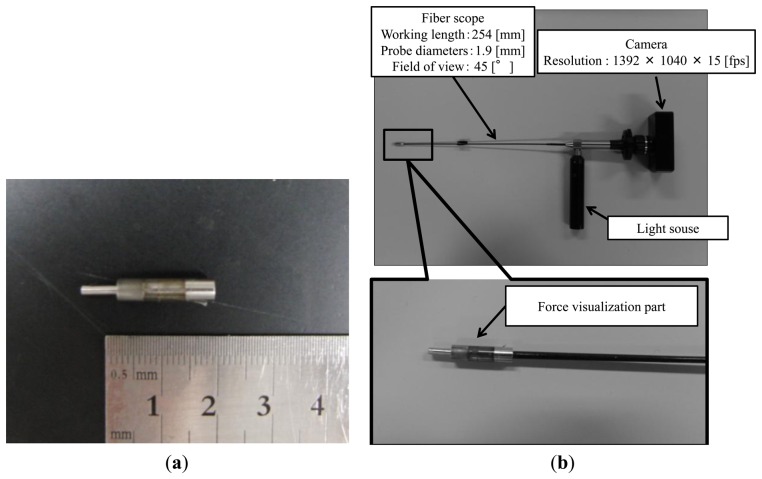
(**a**) Photograph of manufactured force visualization part (**b**) Complete view of developed force sensing system.

The designed force visualization part is shown in Figure 3a. Figure 3b shows a sectional view of the design. A 20-denier panty stocking fabric is installed at region A in Figure 3. Figure 3c shows an overview of the assembly process of the force visualization part, where pins are used for linking the parts. First, part **(IMAGE)** was linked with part **(IMAGE)**, whereas part **(IMAGE)** was linked with part **(IMAGE)**. Second, part **(IMAGE)** was connected to the linked part **(IMAGE)**+**(IMAGE)**. Lastly, the linked parts **(IMAGE)**+**(IMAGE)** and **(IMAGE)**+**(IMAGE)**+**(IMAGE)** were connected. Figure 4a shows a photograph of the manufactured force visualization part. The material used for the visualization part was duralumin, except for the fabric. Figure 4b shows the entire system, including where the force visualization part is attached to the fiberscope. The fiberscope used in this paper is a Super Thin Flexible Borescope (MEDIT INC, Winnipeg, Canada). Its diameter is 1.9 mm, its full length is 254 mm, and its visual field is 45°. The camera (Lu 135, Lumenera Corporation, Ottawa, ON, Canada) is connected to the fiberscope. The specifications (measurement conditions) of the camera are listed in Table 1. The image captured using the Lu 135 can be directly sent to a PC via a USB cable. Note that we investigated the effect of distortion when bending the endoscope and found that there was no change of intensity in the captured image.

## 3. Relationship between Applied Force and Moving Distance of Pin

To derive the magnitude of the applied force from the captured image using the radius of the circular image of the pin, we need to know the relationship between the applied force and the moving distance of the pin due to the applied force. This is because the moving distance determines the size of the circular image of the pin. This relationship depends on the used material itself, and if the material changes, the relationship would also change. As such, this relationship is investigated experimentally.

### 3.1. Experimental Setup and Procedure

**Figure 5.**
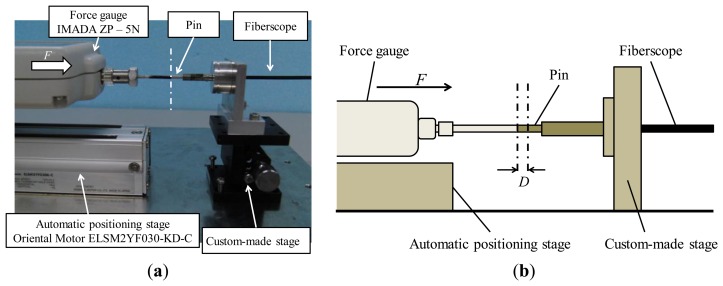
(**a**) Picture of experimental setup (**b**) Image of experimental setup.

Figure 5 shows the experimental setup for the investigation. A force is applied by a force gauge (ZP-5N, IMADA, Toyohashi, Aichi, Japan, resolution: 0.001 N) attached to an automatic positioning stage (ELSM2YF030-KD-C, Oriental Motor, Tokyo, Japan, movement speed: 100 mm/s). A pin with a diameter of 1.9 mm is attached to the tip of the force gauge. This pin is used for pushing the force visualization part attached to the fiberscope. The fiberscope is fixed to a custom-made stage so that the direction of pushing can be controlled appropriately. The position and orientation of the two pins attached to the force gauge and the fiberscope are set up such that their position and orientation are the same.

Because cases involving tissue release are rare, as mentioned above, we focus on the case of pushing tissues. The first step in the experiment was to set up the initial state such that the value shown by the force gauge is 0.000 N while both pins are in contact with each other. Then, the automatic positioning stage was moved in steps of 0.2 mm to push the pin attached to the fiberscope until the total distance moved reached 2.0 mm. At each step, the applied force was measured by the force gauge. This procedure was repeated five times.

### 3.2. Relationship between Applied Force and Pin Movement

**Figure 6.**
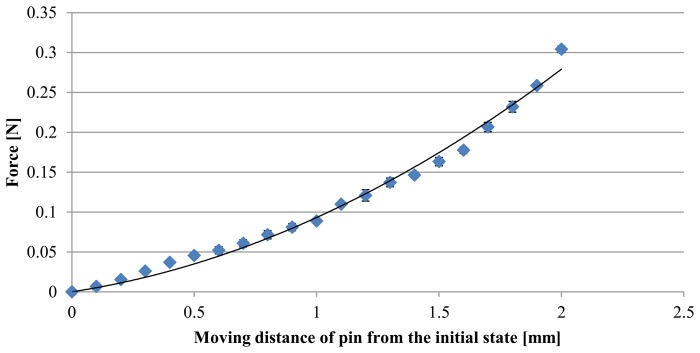
Relationship between applied force and moving distance of pin. Error bars show standard deviation. Note that they are too small to see in some cases.

Figure 6 shows the experimental results for the relationship between the applied force and the moving distance of the pin from the initial state. The diamond symbols indicate the results when the pin is pushing. The error bars show the standard deviation. A regression analysis was performed using second-order polynomial function:

(1) $$F=aD^{2}+bD$$

where *a* = 4.6 × 10^−2^ and *b* = 4.7 × 10^−2^. The coefficients of determination were 0.99. It can be seen that the fitted curve expresses the relationships very well.

The used (knitted) fabric has a reticular pattern and is manufactured using multiple (bended and twisted) strings. If the load is low, the deformation of the fabric is mainly caused by stretching the bended strings. The relationship between load and displacement can then be expressed by a nonlinear function [17,18]. If the load is large, the stings are stretched, and the deformation is then dominated by the stiffness of the stings themselves. In this case, the relationship between load and displacement can be expressed using a linear function. In our case, the maximum value of the applied force was roughly 0.3 N and the load was assumed to be low. In this case, a nonlinear function should be chosen for regression. This is why we used a second-order polynomial function.

## 4. Methodology for Derivation of Force Value from a Captured Image

In this section, the methodology for deriving the magnitude of the applied force from the captured image is described. First, the circular area corresponding to the area of the pin within the elastic fabric is extracted. The magnitude of the applied force is then derived from the radius of the circular area.

### 4.1. Extraction of Circular Image of Pin

**Figure 7.**
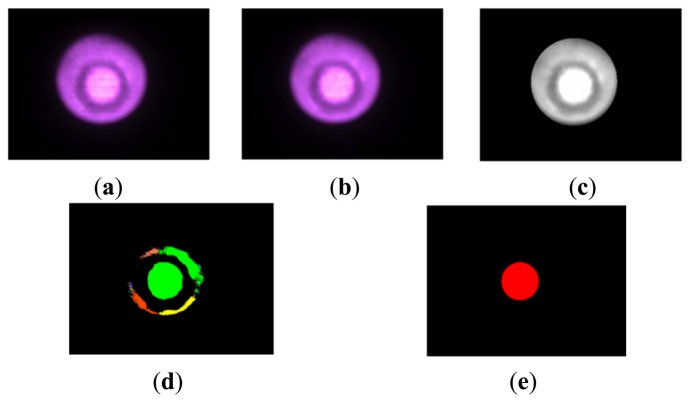
(**a**) Original image (**b**) Smoothing and edge enhancement (**c**) Grayscale image **(d)** Binarizing and selecting area **(e)** Obtained circular area of pin.

Figure 7 shows the procedure and corresponding image at each step. Figure 7a shows the original captured image. The area surrounded by the relatively dark area is the area of the pin with the elastic fabric. We took the following steps for extracting circular area corresponding to the pin area:

➀ Smoothing and edge enhancement: To reduce noise and to clarify the boundary of the pin area, smoothing and edge enhancement are performed. Figure 7b shows the obtained image.
➁ Converting to a grayscale image: As a preprocessing step for binarization, the (color; image is converted to a grayscale image. The obtained image is shown in Figure 7c.
➂ Binarizing and selecting area: To obtain information regarding the radius of the pin area, we binarize the grayscale image. We determine the pin area in the obtained binarized image by searching the area that has the maximum area. Figure 7d shows the obtained image.
➃ Extracting circular area: The largest area (corresponding to the area of the pin with the elastic fabric) is detected, and we derive the minimum circular area including the largest detected area (pin area;. The obtained circular area is shown in Figure 7e. Hereafter, we call this circular area the circular pin image. Finally, we derive the radius of the obtained circular area (circular pin image).

We implemented these processing steps with Halcon (MVTech, Munich, Germany). The computing time is less than 90 ms, and 10 ms without the smoothing procedure. If the processing time in Halcon is less than in conventional C++ programing code, the computing time is considered to be fast enough to display the force information on the monitor (visual image) in real time.

### 4.2. Deviation of Magnitude of Force

**Figure 8.**
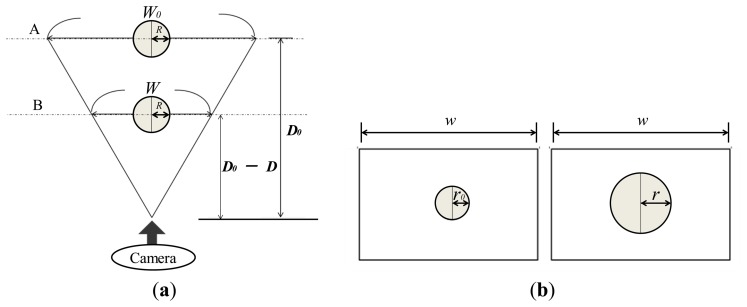
(**a**) Size of object area depends on its location. The object area at location B is larger than that at location A in the image captured by the camera. (**b**) Image captured by the camera when object is located at A (left) and object is located at B (right).

To obtain the relationship between the applied force and the area of the radius of the circular pin image, we consider the relationship between the object area in the image and the actual location of the object. Consider the case shown in Figure 8a where a spherical object with radius, *R*, is captured by a camera. We compare the cases when the distance between the object and the camera is *D*_0_ and *D*_0_-*D. W*_0_ and *W* denote the actual length corresponding to the width (*w*) in the image captured by the camera when the distance is *D*_0_ and *D*_0_-*D*, respectively. Figure 8b shows the image captured by the camera in Figure 8a. The left panel of Figure 8b shows the case when the object is located at a distance *D*_0_ (corresponding to A in Figure 8a). The right panel of Figure 8b shows the case where the object is located at a distance *D*_0_-*D* (corresponding to B in Figure 8a). *r*_0_ and *r* are the apparent radii of the spherical object with a radius of *R* in the captured image.

(2) $$\frac{r_{0}}{r}=\frac{D_{0}-D}{D_{0}}$$

On the other hand, the relationship between pin movement and applied force is given in Equation (1). By substituting Equation (2) into Equation (1), the following relationship is obtained:

(3) $$F=\alpha {\left(\frac{1}{r}\right)}^{2}+\beta \left(\frac{1}{r}\right)+\gamma$$

where:

(4) $$\begin{array}{c} \alpha =aD_{0}^{2}r_{0}^{2}\\ \beta =-(2aD_{0}^{2}r_{0}^{2}+bD_{0}r_{0})\\ \gamma =aD_{0}^{2}+bD_{0} \end{array}$$

From Equation (2), it can be seen that the magnitude of the applied force can be expressed using a second-order polynomial function with respect to the inverse of the radius of the obtained circular pin image.

## 5. Experiment

By performing an experiment for investigating the relationship between the applied force and the radius of the circular pin image, we demonstrate the validity of our approach. In neurosurgery, most of the organs are normally in a steady state. Therefore, we conducted experiments where the target does not move.

The experimental setup is the same as that described in Section 3.1. The procedure is as follows. First, we set up an initial state where the value of the force gauge is 0.000 N while both pins are in contact with each other. Then, the automatic positioning stage, used to push the pin attached to the fiberscope, is moved in steps of 0.02 N. Note that the step size was based upon the magnitude of the applied force. The magnitude was controlled using the automatic positioning stage with a resolution of 0.01 mm, confirming the force value measured by the force gauge. In addition, note that the camera can only catch light from the light source shown in Figure 6. This experiment was repeated five times.

**Figure 9.**
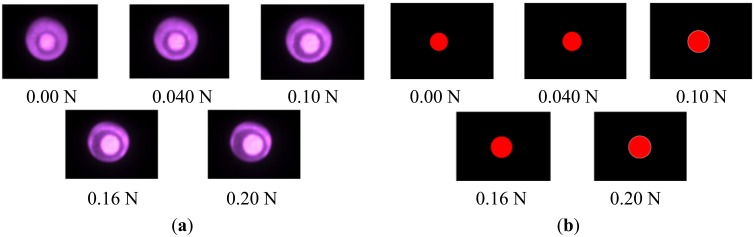
(**a**) Image captured by camera attached to fiberscope. (**b**) Circular pin image.

Figure 9a shows the captured image. The area of the pin increases with the increase in applied force. Figure 9b shows the corresponding circular pin image obtained through the image processing method described in Section 4.1. As described in the previous section, the magnitude of the applied force can be represented by a second-order polynomial function with respect to the inverse of the radius. We can then approximate the relationship with the equation given in Equation (2):

(5) $$F=1.06\times 10^{4}{\left(\frac{1}{r}\right)}^{2}-2.10\times 10^{2}\left(\frac{1}{r}\right)+1.05$$

**Figure 10.**
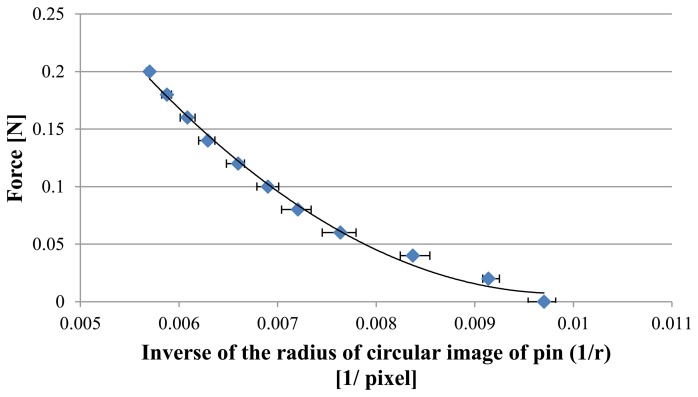
Relationship between applied force and inverse of radius of circular image of pin.

Figure 10 shows the obtained relationship between the applied force and the inverse of the radius of the circular pin image with the obtained fitted curve. The coefficient of determination was 0.99, and the fitted curve accurately represents the relationship. The range of the applied force ranged from 0 to 0.2 N, and the force applied to the elastic fabric was assumed to be small. Therefore, the function given in Equation (4) accurately represented the relationship, and a high coefficient of determination was obtained.

**Figure 11.**
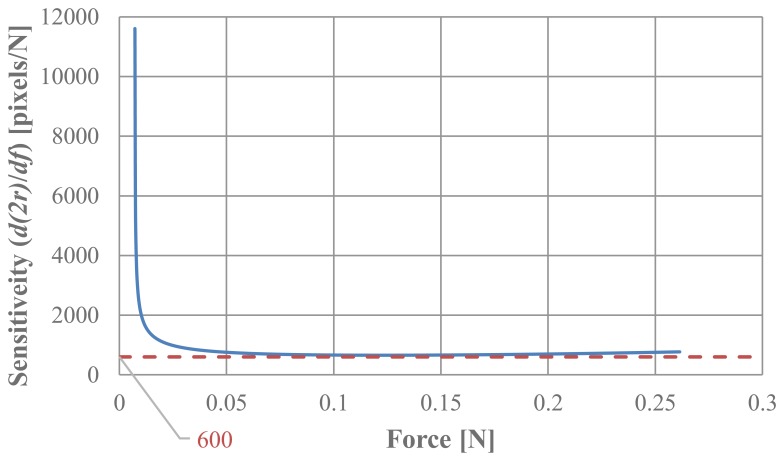
Relationship between sensitivity *d*(2*r*)/*df* and applied force.

Because the relationship given in Equation (4) is nonlinear, the resolution and sensitivity vary with the range. For sensitivity analysis, *d*(2*r*)/*df* was calculated based on Equation (4). Because the minimum amount of change in *r* (radius of the circular pin image) is 0.5 pixels, not *dr*)/*df* but rather *d*(2*r*)/*df* was calculated (2*r* corresponds to the diameter of the circular pin image). Figure 11 shows the obtained relationship between the sensitivity *d*(2*r*)/*df* and applied force. It can be seen that the sensitively is slightly larger than 600 pixels/N when the applied force exceeds 0.05 N, while the sensitivity is very high when the applied force is less than 0.05 N. The sensitivity always exceeded 600 pixels/N, which means one pixel corresponds to 0.0017 N. This minimal value (600 pixels/N) is considered to be the sensitivity of the developed sensor system. It is considered to represent high sensitivity.

**Figure 12.**
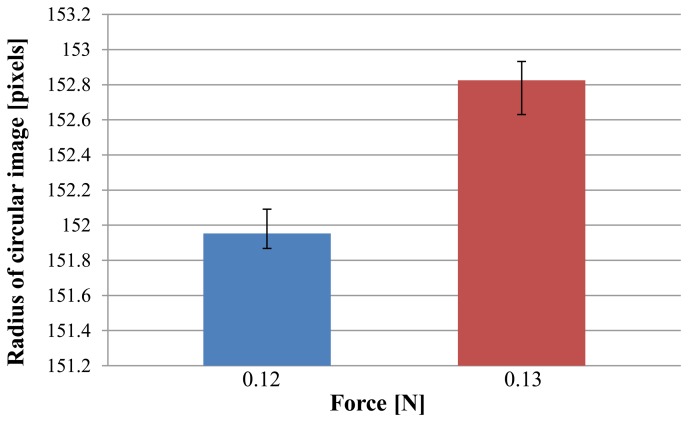
Radius of circular image of pin for 0.12 N and 0.13 N measured five times. Error bars show maximum differences.

From Figure 11 and Equation (4), the sensitivity is minimal at around 0.12–0.13 N. Therefore, we focused on this area to examine the resolution. We measured the radius of the circular pin image for a case of 0.12 N and 0.13 N five times. These results are shown in Figure 12. A *t*-test was applied, and a value of *p* ≪ 0.005 was obtained. This shows that there is a statistically significant difference between the cases of 0.12 N and 0.13 N. This indicates that the resolution is at least less than 0.01 N.

## 6. Conclusions

This paper presents a novel force sensing system that can be used with a thin and small fiberscope/endoscope. We developed a part that transforms force into deformation of a highly elastic fabric. The developed part can be attached to the fiberscope/endoscope, and the deformation can be captured using a camera. By visualizing the force, we eliminated the need for using electrical parts for force sensing. This design also has the advantages of easy sterilization, small size, and low cost. We also described the methods used to derive the magnitude of the applied force from the captured image. We also showed that the force can be measured with high resolution (less than 0.01 N) and high sensitivity (greater than 600 pixels/N).

The goal of developing the system is to enable medical investigation of tissues located in areas deep within organs. By attaching the developed part to a fiberscope, force information can be obtained when visual information might however still be difficult to acquire. A solution to this problem is to use two fiberscopes. One fiberscope is used for force information, whereas the other is used for visual information.

Although the circular pin image can be used for force feedback information, intuitively estimating the force value is difficult owing to the nonlinearity. Future directions in this area could involve the development of an appropriate force feedback system. The presented force sensor can measure forces in only one direction. In situations requiring examinations that involve the release of tissues, hysteresis may need to be considered [19]. We did not check whether the device was waterproof, or consider the measurement of dynamic forces. These issues will also be addressed in future research.

## Figures and Tables

**Table 1.** Specifications of Lumenera Lu 135 Camera.

**Size of Image**	1,392 × 1,040
**Frame Rate**	15 fps
**Exposure Time**	66 ms
**Whole Gain**	2.0 dB
